# Comparison of Manual versus Automated SARS-CoV-2 Rapid Antigen Testing in Asymptomatic Individuals

**DOI:** 10.3390/jcm12227146

**Published:** 2023-11-17

**Authors:** David T. Harris, Nicole Ingraham, Michael Badowski

**Affiliations:** 1 University of Arizona Health Sciences Biorepository, Tucson, AZ 85724, USA; nicoleingraham@arizona.edu (N.I.); badowski@arizona.edu (M.B.); 2 Department of Immunobiology & Medicine, The University of Arizona, Tucson, AZ 85724, USA; 3 Biorepository, The University of Arizona, AHSC 6122, 1501 N Campbell Ave, P.O. Box 245221, Tucson, AZ 85724, USA

**Keywords:** SARS-CoV-2, COVID-19, rapid antigen testing, asymptomatic, diagnostics

## Abstract

The SARS-CoV-2 pandemic has infected more than 770 M people and killed more than 6.9 M persons worldwide. In the USA, as of August 2023, it has infected more than 103 M people while causing more than 1.1 M deaths. During a pandemic, it is necessary to rapidly identify those individuals infected with the virus so that disease transmission can be stopped. We examined the sensitivity of the Quidel Rapid Antigen test on the manual Sofia 2 platform and the Beckman-Coulter antigen test on the automated DxI-800 system for use in screening asymptomatic individuals at the University of Arizona from March through May 2021. A total of 378 asymptomatic subjects along with 176 validation sets of samples in 23 independent experiments were assessed in side-by-side antigen testing using both assays. Nasal swabs and saliva were used as viral sources. Manual testing (Quidel) was compared with automated testing (Beckman) methods for cost and efficiency. Limit dilution of viral antigen spiked samples was performed to determine sensitivity to antigen load by the tests. The results between the two tests were found to be concordant. Both tests were comparable in terms of detecting low numbers of positive subjects in the asymptomatic population. A concordance of 98% was observed between the two tests. Experiments also demonstrated that saliva specimens were an acceptable viral source and produced comparable results for each test. Overall, the two methods were interchangeable.

## 1. Introduction

In the USA, as of August 2023, the SARS-CoV-19 virus has infected more than 103 M people while causing more than 1.1 M deaths [1]. Despite vaccination, these numbers continue to mount, particularly in areas outside of Western Europe and North America without access to vaccines and in parts of the USA with a higher percentage of vaccine-reluctant residents. Waning immunity and the emergence of novel variants also play a role in persistent infections. As the pandemic has persisted for 2+ years now, there is increasing demand to “reopen” cities, workspaces, and schools and university campuses in a bid to return to normal, despite the risks involved. Pandemic fatigue has become commonplace. To achieve a return to normalcy, it is necessary to be able to rapidly identify potentially large numbers of virus-infected individuals so that disease transmission can be stopped. Universities and academic hospitals can play a critical role in this process due to their experience in working with biohazardous specimens, familiarity with large-scale patient recruitment methods, and ability to deploy large-scale testing procedures.

Tracing efforts required to establish the identity of infected persons depend on the rapid identification of the primary infected person. The source of infection must be isolated and quarantined for observation. Testing strategies must return results to those responsible in as short a time as possible in order to limit viral spread [2]. Typically, polymerase chain reaction (PCR) assays have been the gold standard for viral diagnostic testing [3,4], but other testing options are available. Unfortunately, the PCR test is not generally a point-of-care test and often takes 24–72 h to return results, is relatively expensive at scale, has been plagued by reagent scarcity, and may even be too sensitive at times [2,3,4].

Two rapid viral antigen tests have recently become available in response to the above needs. A manual testing strategy is commercially available from Quidel, Inc. (San Diego, CA, USA) to analyze subjects for the presence of the SARS-CoV-2 viral antigen Ag [5] using a fluorescence assay. The assay (Sofia SARS Antigen FIA) detects the presence of antigens produced by a viral infection rather than the presence of the viral genome, which may not be indicative of an active infection. The test reports the result within 15–30 min, can be scaled to perform thousands of tests per day, has US Food and Drug Administration (FDA) Emergency Use Authorization (EUA) for symptomatic subjects, is relatively inexpensive at USD 23/test, and can be performed using self-administered (anterior) nasal swabs. Beckman-Coulter has developed an automated high-throughput viral rapid antigen test using a luminescence assay to detect the presence of the SARS-CoV-2 viral antigen [6]. The Beckman-Coulter test can also be read in 10–30 min after virus extraction, is capable of testing 200 or more samples per hour in an automated fashion, is extremely inexpensive at USD 10/sample, has applied for FDA EUA, and is amenable to the use of both NP (nasopharyngeal) and nasal swabs as biospecimen sources.

Both Quidel and Beckman-Coulter are companies with a history of producing FDA-approved clinical diagnostic tests, lending confidence to purported reliability claims. Both testing devices, the Sofia 2 and the Beckman-Coulter DxI, have the capability to both display results locally to the operator as well as report into cloud applications or include them directly in subject medical records. The sensitivity of both SARS-CoV-2 rapid Ag tests is reported to be 90%+ with near-100% specificity [5] (FDA EUA Application and 5–6), but these results were not based on testing large numbers of “real-world” samples in a large asymptomatic population.

In the present study, we examined the sensitivity of the manual Quidel and the automated Beckman-Coulter rapid Ag tests in side-by-side use in screening asymptomatic individuals in the student and staff community at the University of Arizona from March to May 2021 (to coincide with Spring Break and semester graduation). After test validation [7], a total of 176 validation sets and 378 asymptomatic subjects in 22 independent assessments were performed using both antigen tests side-by-side at the same time with specimens obtained from the same subject. All recruitment and testing procedures were performed by the University of Arizona Health Sciences (UAHS) Biorepository staff at the University of Arizona. In addition to the sensitivity of detection, we also compared the manual versus automated SARS-CoV-2 testing methods for sample throughput, time required for testing, and cost effectiveness (particularly personnel costs).

## 2. Materials and Methods

### 2.1. Subjects

All human subjects in the study were students, faculty, or staff at the University of Arizona. Participation was voluntary. Asymptomatic subjects were asked to volunteer for testing as a screen for return-to-campus activities, and none had symptoms of infection at the time of biospecimen collection. All demographic data were de-identified upon receipt and all data outcomes pertaining to this study were de-identified prior to submission for publication. All collections were performed between March and May 2021 under IRB protocol #2103592492A001 entitled “Novel High-Throughput SARS-CoV-2 Screening” with an expiration date of 22 March 2022. This time period was chosen to coincide with the start of Spring Break at the university and the end of the Spring semester.

### 2.2. COVID-19 Validation Sets

COVID-19 antigen “spiked” swabs were used to validate the Beckman-Coulter and Quidel tests. That is, swabs containing virus antigen were tested in 22 independent experiments using 22 independently spiked swabs. Spiked swab positive controls were supplied by Quidel as part of the normal control samples included with the Sofia SARS antigen FIA tests kits. A different spiked swab was used for each independent experiment and was placed in either VTM (viral transport media) or UTM (Remel M4RT^TM^ Transport Media (The University of Arizona, Tucson, AZ, USA)) prior to virus extraction and testing. Validation sets were tested at multiple twofold dilutions to determine the ability to detect low antigen loads (undiluted, 1:2, 1:4, etc.). 

### 2.3. Biospecimen Collection

Possible SARS-CoV-2 antigens (Ag) containing specimens were collected using nasal swabs as recommended by each test kit manufacturer. Dry anterior nasal collections were self-performed for both nostrils, each with a different nasal swab under laboratory supervision. Both dry swabs were placed in a single barcoded 15 mL conical tube, kept in an insulated cooler to maintain 65–80 °F (18–27 °C), and transported within 60 min of collection to the testing laboratory. The dual swabs were assigned to one of the testing methods with one swab randomly going to the Quidel method and the other swab going to the Beckman-Coulter testing protocol. Swabs used for the Quidel test were manually processed according to the company’s instructions, which include incubation of the dry swab in 200 µL rehydration solution that contains the necessary chemistry for the Sofia fluorescence assay. Dry swabs used for the automated Beckman-Coulter test were placed in 3 mL of UTM buffer and then extracted according to the manufacturer’s instructions. Saliva samples were also collected for comparison using a saline gargle approach which has been validated to be as sensitive as nasal swabs in PCR testing [8].

### 2.4. Viral Antigen Testing

The Quidel Sofia 2 SARS antigen FIA (fluorescent immunoassay) test displays a digital readout which must be manually recorded as positive or negative based on the ratio of relative fluorescence units from the sample being tested to the background fluorescence in an LFT (lateral flow test) assay termed the S/CO (signal versus control) ratio [5]. Ratios 1.0 and above are considered positive results, and ratios can range from 1–600 depending on the level of viral infection (viral load). While higher S/CO values are generally associated with higher antigen loads, these values cannot be considered linearly quantitative. Samples were processed according to the manufacturer’s instructions within 90 min of arrival at the laboratory and analyzed using the Sofia 2 instrument, which included 1 min of incubation in the reconstitution buffer followed by 15 min of development in the testing cassette.

The Beckman-Coulter Access SARS-CoV-2 Antigen Assay (IFU (Copenhagen, Denmark)) was performed according to the manufacturer’s instructions on the DxI-800 platform [6]. Specimens were automatically extracted from the swabs via transfer of 35 µL of Antigen Extraction Solution to a polypropylene tube. Then, 665 µL of specimen from the swab incubated in 3 mL transport buffer (VTM for validation sets or Remel M4RT for asymptomatic screening) was placed into the polypropylene tube. The sample was incubated for at least 10 min (and no more than 6 h) at room temperature. Samples were automatically read using a DxI machine in a luminescence assay to obtain the S/CO ratio (Signal to Cutoff ratio). An integrated computer/software system determined if a sample was positive or negative. Samples with S/CO ratios of 1.0 or above were considered positive.

The limit of detection (LOD) for both assays was estimated to be approximately 1000 viral particles, as determined by previous PCR studies and cycle threshold values [7]. Both rapid antigen tests are based on detection of viral N1 and N2, and detected all viral variants present in the population at the time of the study (alpha, beta, and delta).

## 3. Results

The utility of the individual SARS-CoV-2 rapid viral antigen tests was assessed in two ways: using validation sets of known positive samples and using nasal swabs obtained from subjects that were probably exposed but asymptomatic. The results of testing using viral antigen “spiked” validation samples to assess tests’ sensitivity are shown in Table 1. A total of 17 experiments were performed using the manual Quidel assay with 136 independent samples, while a total of 22 experiments were conducted using the automated Beckman-Coulter assay with 176 independent samples. In these experiments, a limiting dilution was performed using undiluted samples as well as twofold dilutions of the original sample up to a 1:128 dilution to determine the sensitivity to antigen concentration using the different tests. The results between the two testing methods were concordant and in positive agreement. The two tests were observed to be statistically identical up through a 1:64 dilution.

The potential for false positive results (common with rapid antigen tests; [7]) was next examined. It was observed that, based on the nature of the automated Beckman-Coulter assay (a luminescence reaction in solution), false positive results were not observed, in contrast with the occasional false positive result with the manual Quidel fluorescence LFT assay. A false positive result was inferred when one testing method continued to produce a positive result at very high dilutions when the other test did not. It was observed that the Quidel assay produced positive results when UTM versus VTM [9,10] was used as a transport medium, which in fact were false positive results. These results were confirmed by the observation of “positive” results even when the transport medium without virus was tested (see Table 2, Negative Controls). It should be noted that the Quidel Sofia protocol includes its own rehydration solution and that this solution was replaced with UTM media for these negative controls. No false positives were observed with the Quidel tests when the Quidel supplied rehydration solution was used.

We next examined the influence that the sample transportation buffer might have on the testing results in greater detail, as shown in Table 2. Beckman-Coulter recommends the use of UTM for sample transport, while many facilities commonly use VTM for such activities, including sample collection for PCR assays. As shown in Table 2, when using UTM, the Quidel assay produced positive results even at dilutions as high as 1:128 and even when virus was not present in the assay. The Beckman-Coulter test, however, produced comparable results using either UTM or VTM as the transport buffer, although the VTM buffer seemed to result in slightly higher S/CO values.

Both tests were then assessed in 378 asymptomatic subjects, as shown in Table 3, where most, if not all results were expected to be negative in such an asymptomatic population, particularly as extensive vaccination efforts had been underway since January 2021. As shown in Table 3, both tests were comparable in terms of detecting the low number of positive subjects in the asymptomatic population. A concordance of 100% was observed between the two tests in Table 3. 

Finally, the experiments shown in Table 4 analyzed whether saliva specimens (obtained using the saline-gargle approach described in Methods) could be used as an alternate self-administered biospecimen source [8] of nasal swabs. The results showed that both assays produced comparable results using their respective testing methods. A concordance rate of 96% was observed.

## 4. Discussion

Mitigation of a pandemic such as the SARS-CoV-2 pandemic depends on the rapid and reliable testing of infected (symptomatic) and exposed (asymptomatic) individuals. Although PCR testing has been utilized historically in similar circumstances, it does not lend itself to a rapid turnaround of results, hindering the real-time tracking and tracing efforts that are crucial to slowing the spread of a virus. Rapid antigen tests that identify contagious individuals have been developed that are amenable to a mitigation effort. Testing approaches developed by Quidel and Beckman-Coulter are rapid (30–60 min), can be self-collected using anterior nasal swabs, are relatively inexpensive ($10–23/test), and are capable of high-throughput testing (2000 tests or more per day). The Quidel manual test makes use of a fluorescence LFT approach while the automated Beckman-Coulter test uses a luminescence assay in solution. The Quidel approach uses a small, table-top reader and test cartridges into which the biosample is placed manually. This system was originally designed for doctors’ offices and clinics performing single tests at a time. Due to the volume of testing required, we were able to scale up the manual system to perform thousands of tests per day using multiple machines. The Beckman-Coulter approach uses a large free-standing machine that is automated to read sample large batches, also at the level of thousands of tests per day. Automated reporting is supported by both the Quidel and Beckman-Coulter systems. Both manufacturers have applied for FDA approval of their tests, and the reader devices for both companies can perform other types of clinical assessments (e.g., influenza). The Quidel manual approach to large-scale testing requires the utilization of 6–10 machines (at a cost of $1200 per machine) to achieve a daily testing rate of 2000+ tests per day, while the automated Beckman-Coulter approach requires the use of a single machine (at a cost of $125,000). The total machine costs would be $12,500 for the Quidel approach using 10 machines versus $125,000 for the Beckman-Coulter approach using a single machine (which does have a significantly larger physical footprint). The Beckman-Coulter approach requires a smaller technical staff to reach the same processing goals when considering only the operation of the machine. However, for both systems, a very large fraction of the technicians’ time is spent organizing and labeling samples, performing manual sample extractions from the sample collection swabs, then organizing and racking tubes for assay. At this point, whether the samples were read manually with Quidel or automated with Beckman-Coulter, the total technician time was comparable. 

The Biorepository at the University of Arizona Health Sciences deployed a wide-scale biospecimen collection and testing effort during the pandemic in 2020–2021 to assess community infection rates and to implement mitigation efforts. Prior experience working with biohazardous specimens was instrumental in preventing viral infections in the workplace. In addition, familiarity with large-scale patient recruitment methods and attention to detail needed in the tracking of biospecimens and their donors was crucial to organizing mitigation efforts. Finally, the ability to deploy large-scale virus testing procedures needed to confirm the infectious disease status of the biospecimen donors was required to isolate and quarantine those shedding virus and inhibit further viral spread. 

In the current study, we have validated and evaluated two methodologies (manual and automated) side-by-side using nasal samples collected from the same subjects at the same time. We validated the two methods using virus-antigen-positive nasal swabs and evaluated the sensitivity using nasal swabs collected from 378 asymptomatic individuals, a total of 22 independent experiments collected over a course of 4 months. This period of time was chosen as it spanned from Spring Break at the university to the end of the Spring semester, when infections were likely to be more prevalent, albeit undoubtedly influenced by the vaccination efforts underway since January 2021. Significantly, the two methodologies were greater than 98% concordant over the course of this evaluation. If one only examines the agreement of the two tests for neat, undiluted samples (i.e., disregards the limiting dilution samples shown), then the concordance rate was above 99%. The Beckman-Coulter automated test also did not seem to be prone to false positive results (probably due to the nature of the luminescence assay in solution versus a fluorescence methodology in a solid LFT assay) and was amenable to test samples collected in either VTM or UTM, while UTM media produced a high level of false positive results in the Quidel assay (as demonstrated when UTM was used alone for testing without added virus). Thus, testing of asymptomatic individuals should be performed with samples collected in VTM if a transport buffer is required and the exact nature of the downstream assay is uncertain. Significantly, the Beckman-Coulter test seemed to be applicable not only to anterior nasal samples but also to saliva (saline gargle) specimens, as was the Quidel test, which increases the applicability and utility of the two tests and may encourage more individuals to participate in the mitigation efforts. It should be noted that PCR testing was not performed on the samples used in the rapid antigen testing, and thus false positive and false negative results cannot be excluded, but test concordance between the two approaches makes it unlikely.

Overall, sample testing throughout the experiments was comparable between the manual and automated methods. Testing costs were lower for the manual method when tests and machines are considered. However, the automated method should require fewer laboratory personnel, which is a significant cost savings. When all factors are considered, the overall costs are fairly comparable and both methods are capable of performing tasks as needed.

## Figures and Tables

**Table 1:** Biospecimen testing results using manual versus automated SARS-CoV-2 antigen tests.

Results (% Positive Tests)		
Sample Dilution	Quidel (+) Test	Beckman (+) Test
Undiluted	17/17 (100%)	22/22 (100%)
1:2	17/17 (100%)	22/22 (100%)
1:4	17/17 (100%)	22/22 (100%)
1:8	13/17 (76%)	20/22 (91%)
1:16	10/17 (59%)	14/22 (64%)
1:32	7/17 (41%)	9/22 (41%)
1:64	5/17 (29%)	6/22 (27%)
1:128	4/17 (24%)	2/22 (9%)
N	136	176

Independent virus antigen “spiked” samples were tested for the presence of the SARS-CoV-2 viral antigen using both the manual Quidel and the automated Beckman-Coulter rapid antigen tests. Tests were performed with serial dilutions of the spiked swabs to determine test sensitivity to antigen load. Data are presented as the total number of positive results obtained with each rapid antigen test for the total numbers of antigen tests performed at each dilution (e.g., 5 positive tests observed from 17 total tests shown above performed at a 1:64 dilution equals 29% sensitivity of the Quidel assay).

**Table 2:** Effects of UTM vs. VTM biospecimen transport medium on COVID-19 testing.

Media &			Quidel Test			Beckman Test		
Expt.	#Sample	Dilution	Date	Result	S/CO	Result	S/CO	Concordance
21	522	U0	5/2021	pos	3.6	pos	5.86	match
	532	V0	5/2021	pos	20.8	pos	29.61	match
	523	U2	5/2021	pos	2.4	pos	2.94	match
	533	V2	5/2021	pos	10.1	pos	12.08	match
	524	U4	5/2021	pos	1.7	pos	1.42	match
	534	V4	5/2021	pos	6.7	pos	2.4	match
	525	V8	5/2021	pos	1.4	neg	0.77	non-match
	536	U16	5/2021	pos	3.4	pos	1.42	match
	526	V16	5/2021	pos	1.3	neg	0.44	non-match
	527	U32	5/2021	pos	1	neg	0.26	non-match
	537	V32	5/2021	pos	2.9	neg	0.71	non-match
	528	U64	5/2021	pos	1.2	neg	0.17	non-match
	538	V64	5/2021	pos	2.6	neg	0.43	non-match
	529	U128	5/2021	pos	1.3	neg	0.14	non-match
	539	V128	5/2021	pos	2.4	neg	0.26	non-match
23	566	0	5/2021	pos	6.9	pos	10.83	match
	567	2	5/2021	pos	4.2	pos	5.26	match
	568	4	5/2021	pos	2.8	pos	2.84	match
	569	8	5/2021	pos	1.9	pos	1.5	match
	570	16	5/2021	pos	1.7	neg	0.86	non-match
	571	32	5/2021	pos	1.5	neg	0.47	non-match
	572	64	5/2021	pos	1.5	neg	0.3	non-match
	573	128	5/2021	pos	1.4	neg	0.21	non-match
Negative Controls								
	540	NC	5/2021	pos	2.3	neg	0.09	non-match
	530	NC	5/2021	pos	1.1	neg	0.09	non-match
	531	NC	5/2021	pos	1	neg	0.09	non-match
	591	NC1	5/2021	neg	0.9	neg	0.1	match
	600	NC2	5/2021	pos	1	neg	0.09	non-match

Biospecimen antigen testing using virus-spiked nasal swabs was performed using both the manual Quidel and the automated Beckman-Coulter methodologies as detailed in Materials and Methods. A total of 136 independent samples were examined overall. Two representative experiments are shown to demonstrate the effect of biospecimen transport medium on testing results. Samples were analyzed by limiting dilution using twofold dilutions ranging from undiluted (0 dilution) to 1:128 dilutions. Pos indicates a positive result while neg indicates a negative result on either test based on S/CO values. The same sample extracted in either UTM (U) or VTM (V) was examined in Expt. #21. Expt. #23 was performed using UTM only. Sample results for the two tests were examined for concordance. The red text in the table indicate when a lack of matching (concordance) was observed. NC indicates control experiments wherein samples without virus were tested.

**Table 3:** Testing results using asymptomatic subjects and two different SARS-CoV-2 antigen tests.

Number of Samples		
	Beckman (+)	Beckman (−)
Quidel (+)	5	0
Quidel (−)	0	373

Biospecimens were collected from a total of 378 asymptomatic subjects and tested for COVID-19 using the manual Quidel and the automated Beckman-Coulter SARS-CoV-2 rapid antigen tests simultaneously. Numbers in each cell indicate the total number of subject biospecimens testing positive or negative for the two tests.

**Table 4:** SARS-CoV-2 antigen testing results when using manual or automated testing methods and saline-gargle saliva biospecimens collected from asymptomatic subjects.

Expt. #	Sample #	Date	Quidel Result	S/CO	Beckman Result	S/CO	Concordance
2	33	3/2021	pos	2.1	pos	12.86	match
	34	3/2021	neg	0.4	neg	0.13	match
	35	3/2021	neg	0.2	neg	0.1	match
	36	3/2021	neg	0.2	neg	0.1	match
	37	3/2021	neg	0.8	pos	3.61	non-match
	38	3/2021	neg	0.4	neg	0.48	match
	39	3/2021	neg	0.3	neg	0.31	match
	40	3/2021	neg	0.2	neg	0.15	match
	41	3/2021	neg	0.4	neg	0.19	match
	42	3/2021	neg	0.3	neg	0.14	match
	43	3/2021	neg	0.4	neg	0.11	match
	44	3/2021	neg	0.3	neg	0.13	match
3	45	3/2021	pos	3.5	pos	21.39	match
	46	3/2021	neg	0.5	neg	0.14	match
	47	3/2021	neg	0.3	neg	0.12	match
	48	3/2021	neg	0.4	neg	0.1	match
	49	3/2021	pos	1.6	pos	7.87	match
	50	3/2021	neg	0.8	neg	0.8	match
	51	3/2021	neg	0.6	neg	0.26	match
	52	3/2021	neg	0.5	neg	0.2	match
	53	3/2021	neg	0.5	neg	0.25	match
	54	3/2021	neg	0.5	neg	0.19	match
	56	3/2021	neg	0.4	neg	0.14	match

Rapid antigen testing using saline-gargle saliva biospecimens obtained from asymptomatic individuals was performed using both the manual Quidel and the automated Beckman methodologies as detailed in Materials and Methods. A total of 23 independent samples were examined in two independent experiments. Samples were analyzed undiluted. Pos indicates a positive result while neg indicates a negative result on either test based on S/CO cutoff values. Sample results were examined for concordance. The text in red in the table indicate when a lack of matching (concordance) was observed. Concordance was observed for 22 of 23 individuals.

## Data Availability

All data shown in this study are available upon request.

## Abbreviations

Ag	antigen
M	million
PCR	polymerase chain reaction
FDA	food and drug administration
EUA	emergency use authorization
NP	nasal pharyngeal
LFT	lateral flow test
UTM	universal transport medium
VTM	viral transport medium
